# ASSOCIATION BETWEEN THE SCREEN TIME AND THE CARDIORESPIRATORY FITNESS WITH THE PRESENCE OF METABOLIC RISK IN SCHOOLCHILDREN

**DOI:** 10.1590/1984-0462/2020/38/2019134

**Published:** 2020-06-05

**Authors:** João Francisco de Castro Silveira, Cláudia Daniela Barbian, Leandro Tibiriçá Burgos, Jane Dagmar Pollo Renner, Dulciane Nunes Paiva, Cézane Priscila Reuter

**Affiliations:** a Universidade de Santa Cruz do Sul, Santa Cruz do Sul, RS, Brazil.

**Keywords:** Cardiorespiratory fitness, Sedentary behavior, Child health, Adolescent health, Aptidão cardiorrespiratória, Estilo de vida sedentário, Saúde da criança, Saúde do adolescente

## Abstract

**Objective:**

To verify the association between screen time and cardiorespiratory fitness with the presence of metabolic risk in schoolchildren in an isolated and clustered manner.

**Methods:**

Cross-sectional study with 1.200 schoolchildren from Santa Cruz do Sul-RS. Screen time and cardiorespiratory fitness were evaluated. The continuous metabolic risk score was calculated by summing the Z score of the waist circumference, systolic blood pressure, glucose, triglycerides, total cholesterol, low-density lipoprotein (LDL-C) and high-density lipoprotein (HDL-C).

**Results:**

Children (34.3%) and adolescents (48.2%) had high screen time, while 44.3% of the children and 53.3% of the adolescents were unfit in relation to cardiorespiratory fitness. Regarding the relation of screen time/cardiorespiratory fitness, 14.7% of the children and 26.9% of the adolescents presented high screen time and low levels of cardiorespiratory fitness. The presence of metabolic risk was shown in children (17.1%) and adolescents (14.7%). The presence of metabolic risk was directly associated with low levels of cardiorespiratory fitness in children and adolescents. When analyzed in clusters, the metabolic risk in children was 11% more prevalent in subjects with low screen time/unfit and 12% in subjects with high screen time/unfit, whereas in adolescents, the prevalence of metabolic risk was also higher in those with low screen time/unfit (8%) and high screen time/unfit (7%).

**Conclusions:**

The presence of metabolic risk in children and adolescents was associated with low levels of cardiorespiratory fitness, independent of screen time, in an isolated or clustered manner.

## RESUMO

**Objetivo:**

Verificar a associação entre tempo de tela e aptidão cardiorrespiratória, de forma isolada e agrupada, e a presença de risco metabólico em escolares.

**Métodos:**

Estudo transversal com 1.200 escolares de Santa Cruz do Sul (RS). Foram avaliados o tempo de tela e a aptidão cardiorrespiratória. Foi calculado o escore de risco metabólico por meio da soma do escore Z, da circunferência da cintura, da pressão arterial sistólica, da glicose, dos triglicerídeos, do colesterol total, do colesterol da lipoproteína de baixa densidade (LDL) e do colesterol da lipoproteína de alta densidade (HDL).

**Resultados:**

Crianças (34,3%) e adolescentes (48,2%) apresentaram elevado tempo de tela, enquanto 44,3% das crianças e 53,3% dos adolescentes foram inaptos no tocante à aptidão cardiorrespiratória. Na relação tempo de tela/aptidão cardiorrespiratória, 14,7% das crianças e 26,9% dos adolescentes exibiram elevado tempo de tela e baixos níveis de aptidão cardiorrespiratória. A presença de risco metabólico foi evidenciada em crianças (17,1%) e em adolescentes (14,7%). A presença de risco metabólico esteve associada diretamente com baixos níveis de aptidão cardiorrespiratória nas crianças e nos adolescentes no que se refere às variáveis de forma isolada. Quando analisado de modo agrupado, o risco metabólico em crianças foi 11% mais prevalente em sujeitos com baixo tempo de tela/inaptos e 12% em sujeitos com elevado tempo de tela/inaptos, enquanto em adolescentes a prevalência de risco metabólico também foi superior nos escolares com baixo tempo de tela/inaptos (8%) e elevado tempo de tela/inaptos (7%).

**Conclusões:**

A presença de risco metabólico em crianças e adolescentes esteve associada com baixos níveis de aptidão cardiorrespiratória, independentemente do tempo de tela, tanto de modo isolado quanto agrupado.

## INTRODUCTION

The American College of Sports Medicine^1^ recommends that in order to guarantee health benefits children and adolescents between six and 17 years of age should perform at least 60 minutes of moderate to intense aerobic physical activity daily. Among these benefits, there is an improvement in metabolic risk factors, however a systematic review study^2^ reports that young Brazilians are characterized by adopting health risk behaviors, such as physical inactivity, sedentary behaviors and inadequate nutrition, which can represent a serious threat to the current and future health of young people.^3^

A systematic review^4^ that analyzed sedentary behavior and health indicators showed that individuals who spend longer periods in front of television are more likely to develop dysfunctions. Other authors^5^ ^,^ ^6^ ^,^ ^7^ state that the prevalence of metabolic disorders in adolescents is high when, in addition to being inactive, they have low levels of cardiorespiratory fitness (CRF), which in itself is already considered an indicator of health risk.

The role of CFR in promoting metabolic health is widespread in the literature. This parameter has often been used as a reference in research, as it plays an important role in the definition of metabolic health in children and adolescents.^8^ However, despite the vast evidence in the literature that show that sedentary behavior and CRF are metabolic risks when verified in an isolated way, there are few studies on the role of the relationship between screen time and CRF in a clustered manner with the appearance of metabolic changes in the children and youth population.

Thus, even if CRF and sedentary behavior are recognized as factors associated with the presence of risk factors for the development of metabolic disorders when analyzed separately, studies that seek associations between CRF and sedentary behavior are necessary, such as screen time (ST), in a clustered manner, and the presence of metabolic risk in children and adolescents. After all, it is assumed that the adoption of behaviors characterized as low health risk, such as, less time spent in front of screens, in addition to the diagnosis of good CRF levels, assumes an important role in the definition of children’s metabolic health. It is also important to highlight that this population has several metabolic changes, especially in obesity, blood pressure and lipid indicators, ^9^ however the traditional criteria only identify only one tenth of these subjects at risk.^10^

Thus, the continuous metabolic risk score (cMetS) has been widely used in international studies,^8^ ^,^ ^11^ but it is still little explored in the Brazilian child and adolescent population. Given the above, the present study aimed to assess the association between ST and CRF, both in isolation and in a clustered manner, and the presence of metabolic risk in schoolchildren.

## METHOD

A Cross-sectional study conducted with 1,200 children and adolescents from Santa Cruz do Sul (RS), enrolled as students in public and private schools. This research is part of the Schoolchildren’s Health Project, developed at the University of Santa Cruz do Sul and approved by the Ethics Committee in Research with Human Beings, under opinion number 714.216, and Certificate of Presentation for Ethical Appreciation (CAAE) number 31576714.6.0000.5343. The parents and/or guardians of the students authorized the student´s participation by signing the Free and Informed Consent Form (ICF). The students also signed the Term of Assent.

The study was carried out in 25 randomly selected schools in the municipality, which has a total of 50 registered schools and a population of 17,688 students. The data collected represents the entire municipality, considering the population density of schoolchildren in each region (center, north, south, east and west), urban and rural areas. The sample power calculation was performed using the G*Power 3.1 program (Heinrich-Heine-Universität, Düsseldorf, Germany).^12^

In view of the statistical test applied (Poisson regression) to assess the one-tailed outcome (presence versus absence of metabolic risk), the test power (1−β) = 0.95 was used, the significance level of α=0.05, the effect size of 20% (Exp β1=1.2) and the metabolic risk rate estimated at 15% (Base rate exp(β0)=0.15). Based on this calculation and the notes by Faul et al.,^12^ the minimum sample size of 1,013 subjects was estimated.

**Figure 1:**
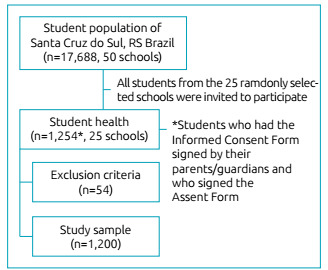
Flow chart of the sample selection.

All students from the 25 schools were invited to participate in the study by sending the informed consent form and the assent form. The initial sample included the presence of 1,254 students, who received informed consent from their parents and/or guardians and who also signed the assent form, however 54 subjects who did not present the data collected in full (CRF test, ST questionnaire or blood collection) were excluded. The sample selection is detailed in Figure 1.

The subjects’ ST was self-reported by completing an adapted questionnaire^13^, and at a later point this variable was classified according to the parameters of the American Academy of Pediatrics:^14^ short time in front of the screen (<2 hours daily) or long time in front of the screen (≥2 hours daily). The evaluation of the CRF was carried out using the 6-minute running and walking test, in which the distance covered by the student (in meters) was used for further categorization, as recommended by *Projeto Esporte Brasil*,^15^: apt for good levels of CRF or unfit for low CRF levels. The ST and CRF variables were grouped together which gave rise to four categories:

- Low screen time: fit.
- Low screen time: unfit.
- High screen time: fit.
- High screen time: unfit.

For the metabolic risk assessment, the cMetS was calculated by adding the Z score of the following parameters: waist circumference, systolic blood pressure, glucose, triglycerides, total cholesterol, low density lipoprotein (LDL) cholesterol and cholesterol of high density lipoprotein (HDL) (this multiplied by -1, for indicating an inverse relationship with cardiovascular risk factors). The data were expressed in a continuous and categorized way, understanding cMetS values above 1 as metabolic risk.^16^

Data analysis was performed using the Statistical Package for the Social Sciences (SPSS) v. 23.0 (IBM, Armonk, NY, United States). Descriptive statistics (absolute and relative frequency; mean and standard deviation) were used to characterize the sample. To assess the association between the independent variables (ST, CRF and ST/CRF ratio) and the outcome (presence of metabolic risk), Poisson regression was used, using the prevalence ratio (PR) values and 95% confidence intervals (95% CI). P values <0.05 were considered significant.

## RESULTS

Table 1 shows the descriptive characteristics of the evaluated students. It is observed that both children (34.3%) and adolescents (48.2%) had a high ST, while 44.3% of children and 53.3% of adolescents were considered unfit according to CRF. When the ST/CRF ratio was analyzed in a clustered manner, 14.7% of children and 26.9% of adolescents exhibited high ST and low levels of CRF. The presence of metabolic risk was evidenced in 17.1% of children and in 14.7% of adolescents.

**Table 1:** Characterization of the evaluated students.

	Children	Adolescent
	n (%)	n (%)
Sex		
Male	161 (49.2)	384 (44.0)
Female	166 (50.8)	489 (56.0)
School network		
Municipal	140 (42.8)	350 (40.1)
State	163 (48.9)	471 (54.0)
Private	24 (7.3)	52 (6.0)
Residential area		
Center	65 (19.9)	200 (22.9)
Periphery	160 (48.9)	332 (38.0)
Countryside	102 (31.2)	341 (39.1)
Socioeconomic class		
A and B	169 (51.7)	477 (54.6)
C	149 (45.6)	370 (42.4)
D and E	9 (2.8)	26 (3.0)
Screen time		
Low screen time	215 (65.7)	452 (51.8)
High screen time	112 (34.3)	421 (48.2)
Cardiorespiratory fitness		
Fit	182 (55.7)	408 (46.7)
Unfit	145 (44.3)	465 (53.3)
ST/CRF Ratio		
Low ST/fit	118 (36.1)	222 (25.4)
Low ST/unfit	97 (29.7)	230 (26.3)
High ST/fit	64 (19.6)	186 (21.3)
High ST/unfit	48 (14.7)	235 (26.9)
Metabolic risk		
Absent	271 (82.9)	745 (85.3)
Present	56 (17.1)	128 (14.7)

ST: screen time; CRF: cardiorespiratory fitness.

Table 2 shows that the presence of metabolic risk was directly associated with low levels of CRF both in children (PR=1.09;95% CI1.01-1.17) and in adolescents (PR=1.08; 95% CI 1.04-1.13), with no association regarding ST.

**Table 2:** Prevalence ratio for the presence of metabolic risk according to screen time and of cardiorespiratory fitness levels in isolation in children and adolescents* .

	Presence of metabolic risk	p-value**
	PR (95% CI)	
Children		
Screen time		
Low ST	1	
High ST	1.04 (0.97-1.12)	0.271
Cardiorespiratory fitness		
Fit	1	0.130
Unfit	1.09 (1.01-1.17)	0.025
Adolescents		
Screen time		
Low ST	1	
High ST	0.99 (0.95-1.03)	0.645
Cardiorespiratory fitness		
Fit	1	0.667
Unfit	1.08 (1.04-1.13)	<0.001

*All values ​​were obtained using Poisson Regression considering the presence *versus* the absence of metabolic risk, and the analyzes were adjusted for sex, socioeconomic level, school network and region of residence ; **significant values ​​for p <0.05; PR: prevalence ratio; 95% CI: 95% confidence interval; ST: screen time; CRF: cardiorespiratory fitness.

The presence of metabolic risk, according to the ST/CRF ratio, is shown in Table 3. It appears that the metabolic risk in children was 11% more prevalent in subjects with low ST/unfit and 12% in subjects with high ST/unfit, while in adolescents the prevalence of metabolic risk was also higher in students with low ST/unfit (8%) and high ST/unfit (7%).

**Table 3:** Prevalence ratio for the presence of metabolic risk according to the screen time/ cardiorespiratory fitness ratio, in children and adolescents* .

	Presence of metabolic risk	p-value**
	PR (95% CI)	
Children		
ST/CRF ratio		
Low ST/fit	1	
Low ST/unfit	1.11 (1.02-1.21)	0.013
High ST/fit	1.08 (0.98-1.18)	0.130
High ST/unfit	1.12 (1.00-1.125)	0.048
Adolescents		
ST/CRF ratio		
Low ST/fit	1	
Low ST/unfit	1.08 (1.02-1.14)	0.011
High ST/fit	0.99 (0.94-1.04)	0.667
High ST/unfit	1.07 (1.01-1.13)	0.020

*All values ​​were obtained using Poisson Regression considering the presence *versus* the absence of metabolic risk, and the analyzes were adjusted for sex, socioeconomic level, school network and region of residence; **significant values ​​for p <0.05; PR: prevalence ratio; 95% CI: 95% confidence interval; ST: screen time; CRF: cardiorespiratory fitness.

## DISCUSSION

This research evaluated the associations between ST and CRF, both in isolation and in a clustered manner, and the presence of metabolic risk in children and adolescents, emphasizing that such risk was associated, in both situations, with low levels of CRF, regardless of ST. Although there is a shortage of studies in the literature that demonstrate a significant association between the ST/CRF ratio and metabolic changes in children and adolescents’ populations, some evidence is consistent with the results of the present study.

One study^17^ assessed whether adolescents with satisfactory CRF levels and low rates of time spent in front of screens had a better metabolic profile compared to those with low CRF levels and long screen times. The results obtained by the referred authors indicated that individuals with low CRF and high ST were three times more likely to have a negative metabolic profile.

A cross-sectional study^18^ that assessed CRF and lifestyle factors, among other factors, including time spent in front of television, computer and video game, found that adolescents who spent two or more hours in front of screens had worse CRF levels. Supposedly, ST would act as an influencer of CRF levels, i.e., the longer the time spent in front of the screens, the lower the CRF indexes. This is justified by the fact that the more time spent in front of screens, the lower the levels of physical activity.^19^

Another study^20^ evaluated changes in sedentary behavior, including ST, moderate to intense physical activities, CRF and metabolic risks, and after ten years of research it concluded that the adoption of moderate to intense physical activities combined with decreasing levels of sedentary behaviors results in positive changes in CRF and are combined with positive changes in metabolic health. However, these facts do not explain why CRF is associated, regardless of ST, with the presence of metabolic risk.

If, on the one hand, the literature is scarce in assessing the presence of metabolic risk with the ST/CRF ratio, several studies expose the relationship between the metabolic risk and the ST and CRF variables, in isolation. Some authors have indicated that different types of sedentary behavior can have many consequences on various health indicators. Among the health indicators assessed in a systematic review study, ^21^ it was found that low levels of physical activity and CRF and high ST were directly linked to the development of metabolic changes in adolescents. In another systematic review, ^4^ the longer duration of time spent watching television was associated with an unfavorable health profile.

The adoption of these behaviors characterizes childhood and adolescence as a sensitive period for the development of metabolic syndrome in mid-adult life and adds that the time spent in front of television must be reduced as early as childhood, before it becomes a chronic behavior,^22^ considering that prolonged periods of time in front of screens during leisure time in adolescence and the increase in the daily frequency of these behaviors are associated with the appearance of cardiovascular risk factors in early adulthood.^23^ Such evidence corroborates the claims that subjects who aim to reduce time in front of screens in addition to the practice of regular physical exercises during childhood and adolescence are more likely to have better metabolic health as adults.^24^ ^,^ ^25^

Regarding the isolated CRF levels, with the presence of metabolic risk, the inverse association between both factors is widely disseminated in the literature. In this sense, the data obtained in the present study corroborate the conclusions of authors^26^ who observed associations between low levels of CRF and the presence of metabolic risks in adolescents in southern Brazil and similar results found in European adolescents.^27^ Other authors^28^ still report that the development of metabolic risks in schoolchildren increases when they are overweight or obese in addition to having low levels of CRF.

Considering the association of CRF, regardless of ST, with the presence of metabolic risk in both analyzes, it is possible to consider CRF as a vital sign of metabolic health in childhood and adolescence. According to Després,^29^ the CRF should still be a priority in practical and public health interventions. However, in addition to the CRF taking a preventive role in the possible appearance of metabolic changes, changes in the adopted behavioral habits are also necessary in order to prevent future unfavorable outcomes to good health conditions, according to the data discussed in this study, since lifestyle habits including sedentary behavior, increased consumption of soft drinks and/or sweetened beverages and physical inactivity below the minimum levels for physical activity, in addition to other factors such as gender, socioeconomic levels and excess of adipose tissue, are determining factors in the definition of good or bad CRF and, consequently, metabolic health levels.^30^

The present study demonstrated that the presence of metabolic risk in both children and adolescents was associated with low levels of CRF regardless of ST and the method (clustered or separated) used for verification. Some strengths can be highlighted in the present study. First, the use of the same professionals during the data collection process, in order to restrict errors and avoid confounding factors; the sample size is representative for the population studied; and, finally, the affirmation of the CRF, once again, as an important marker of health definition in children and youth populations, justifying the importance of developing interventions focused on improving the levels of physical fitness, especially in relation to CRF.

However, this study also has some limitations: due to the cross-sectional design, it is not possible to establish the cause and effect impact between the variables. Thus, it is suggested that future studies seek to verify longitudinal associations, as well as the role played by CRF in the development of metabolic changes. Another limitation of this study is found in the fact that the ST levels were self-reported by the students through questionnaires, which can cause bias when classifying the ST levels, since only what has been reported is considered.
