# ERRATUM

**DOI:** 10.1590/1984-0462/2020/38/2019134erratum

**Published:** 2020-07-29

In the manuscript “Association between the screen time and the cardiorespiratory fitness with the presence of metabolic risk in schoolchildren”, DOI: http://dx.doi.org/10.1590/1984-0462/2020/38/2019134, published in the Rev. Paul. pediatr. [Internet]. 2020;38:e2019134. Epub June 05, 2020, page 6-7.

**Where it reads:**

11. Stavnsbo M, Resaland GK, Anderssen SA, Steene-Johannessen J, Domazet SL, Skrede T, et al. Reference values for cardiometabolic risk scores in children and adolescents: suggesting a common standard. Atherosclerosis. 2018;278:299-306. https://doi.org/10.1016/j.atherosclerosis.2018.10.003

11. Faul F, Erdfelder E, Buchner A, Lang AG. Statistical power analyses using G*Power 3.1: Tests for correlation and regression analyses. Behav Res Methods. 2009;41:1149-60. https://doi.org/10.3758/BRM.41.4.1149

12. Barros MV, Nahas MV. Medidas da atividade física: teoria e aplicação em diversos grupos populacionais. Londrina: Midiograf; 2003.

13. American Academy of Pediatrics. Committee on Public Education. American Academy of Pediatrics: children, adolescents, and television. Pediatrics. 2001;107:423-6. https://doi.org/10.1542/peds.107.2.423

14. Projeto Esporte Brasil [homepage on the Internet]. Manual 2015 [cited 2017 Jul 17]. Available from: https://www.ufrgs.br/proesp/

15. Andersen LB, Harro M, Sardinha LB, Froberg K, Ekelund U, Brage S, et al. Physical activity and clustered cardiovascular risk in children: a cross-sectional study (The European Youth Heart Study). Lancet. 2006;368:299-304. https://doi.org/10.1016/S0140-6736(06)69075-2

16. Mota J, Santos R, Moreira C, Martins C, Gaya A, Santos MP, et al. Cardiorespiratory fitness and TV viewing in relation to metabolic risk factors in Portuguese adolescents. Ann Hum Biol. 2013;40:157-62. https://doi.org/10.3109/03014460.2012.752524

17. Gonçalves EC, Silva DA. Factors associated with low levels of aerobic fitness among adolescents. Rev Paul Pediatr. 2016;34:141-7. http://dx.doi.org/10.1016/j.rppede.2015.06.025

18. Sandercock GR, Ogunleye AA. Screen time and passive school travel as independent predictors of cardiorespiratory fitness in youth. Prev Med. 2012;54:319-22. https://doi.org/10.1016/j.ypmed.2012.03.007

19. Knaeps S, Bourgois JG, Charlier R, Mertens E, Lefevre J, Wijndaele K. Ten-year change in sedentary behavior, moderate-to-vigorous physical activity, cardiorespiratory fitness and cardiometabolic risk: independent associations and mediation analysis. Br J Sports Med. 2016;52:1063-8. http://dx.doi.org/10.1136/bjsports-2016-096083

20. Oliveira RG, Guedes DP. Physical activity, sedentary behavior, cardiorespiratory fitness and metabolic syndrome in adolescents: Systematic review and meta-analysis of observational evidence. PLoS One. 2016;11:e0168503. https://doi.org/10.1371/journal.pone.0168503

21. Wennberg P, Gustafsson PE, Howard B, Wennberg M, Hammarström A. Television viewing over the life course and the metabolic syndrome in mid-adulthood: a longitudinal population-based study. J Epidemiol Community Health. 2014;68:928-33. https://doi.org/10.1136/jech-2013-203504

22. Grøntved A, Ried-Larsen M, Møller NC, Kristensen PL, Wedderkopp N, Froberg K, et al. Youth screen-time behavior is associated with cardiovascular risk in young adulthood: the European Youth Heart Study. Eur J Prev Cardiol. 2014;21:46-56. https://doi.org/10.1177/2047487312454760

23. Wennberg P, Gustafsson PE, Dunstan DW, Wennberg M, Hammarström A. Television viewing and low leisure-time physical activity in adolescence independently predict the metabolic syndrome in mid-adulthood. Diabetes Care. 2013;36:2090-7. https://doi.org/10.2337/dc12-1948

24. Silva DR, Werneck AO, Collings PJ, Fernandes RA, Barbosa DS, Rongue ER, et al. Physical activity maintenance and metabolic risk in adolescents. J Public Health (Oxf.). 2018;40:493-500. https://doi.org/10.1093/pubmed/fdx077

25. Silva D, Werneck AO, Collings P, Tomeleri CM, Fernandes RA, Rongue E, et al. Cardiorespiratory fitness is related to metabolic risk independent of physical activity in boys but not girl from Southern Brazil. Am J Hum Biol. 2016;28:534-8. https://doi.org/10.1002/ajhb.22826

26. Artero EG, Ruiz JR, Ortega FB, España-Romero V, Vicente-Rodríguez G, Molnar D, et al. Muscular and cardiorespiratory fitness are independently associated with metabolic risk in adolescents: the HELENA study. Pediatr Diabetes. 2011;12:704-12. https://doi.org/10.1111/j.1399-5448.2011.00769.x

27. Todendi PF, Valim AR, Reuter CP, Mello ED, Gaya AR, Burgos MS. Metabolic risk in schoolchildren is associated with low levels of cardiorespiratory fitness, obesity, and parents’ nutritional profile. J Pediatr (Rio J). 2016;92:388-93. http://dx.doi.org/10.1016/j.jped.2015.10.007

28. Després JP. Physical activity, sedentary behaviours and cardiovascular health: when will cardiorespiratory fitness become a vital sign? Can J Cardiol. 2016;32:505-13. https://doi.org/10.1016/j.cjca.2015.12.006

29. Gonçalves EC, Silva AS, Nunes HE. Prevalence and factors associated with low aerobic performance levels in adolescents: a systematic review. Curr Pediatr Rev. 2015;11:56-70. https://doi.org/10.2174/1573396311666150501003435

**It should read:**

11. Stavnsbo M, Resaland GK, Anderssen SA, Steene-Johannessen J, Domazet SL, Skrede T, et al. Reference values for cardiometabolic risk scores in children and adolescents: suggesting a common standard. Atherosclerosis. 2018;278:299-306. https://doi.org/10.1016/j.atherosclerosis.2018.10.003

12. Faul F, Erdfelder E, Buchner A, Lang AG. Statistical power analyses using G*Power 3.1: Tests for correlation and regression analyses. Behav Res Methods. 2009;41:1149-60. https://doi.org/10.3758/BRM.41.4.1149

13. Barros MV, Nahas MV. Medidas da atividade física: teoria e aplicação em diversos grupos populacionais. Londrina: Midiograf; 2003.

14. American Academy of Pediatrics. Committee on Public Education. American Academy of Pediatrics: children, adolescents, and television. Pediatrics. 2001;107:423-6. https://doi.org/10.1542/peds.107.2.423

15. Projeto Esporte Brasil [homepage on the Internet]. Manual 2015 [cited 2017 Jul 17]. Available from: https://www.ufrgs.br/proesp/

16. Andersen LB, Harro M, Sardinha LB, Froberg K, Ekelund U, Brage S, et al. Physical activity and clustered cardiovascular risk in children: a cross-sectional study (The European Youth Heart Study). Lancet. 2006;368:299-304. https://doi.org/10.1016/S0140-6736(06)69075-2

17. Mota J, Santos R, Moreira C, Martins C, Gaya A, Santos MP, et al. Cardiorespiratory fitness and TV viewing in relation to metabolic risk factors in Portuguese adolescents. Ann Hum Biol. 2013;40:157-62. https://doi.org/10.3109/03014460.2012.752524

18. Gonçalves EC, Silva DA. Factors associated with low levels of aerobic fitness among adolescents. Rev Paul Pediatr. 2016;34:141-7. http://dx.doi.org/10.1016/j.rppede.2015.06.025

19. Sandercock GR, Ogunleye AA. Screen time and passive school travel as independent predictors of cardiorespiratory fitness in youth. Prev Med. 2012;54:319-22. https://doi.org/10.1016/j.ypmed.2012.03.007

20. Knaeps S, Bourgois JG, Charlier R, Mertens E, Lefevre J, Wijndaele K. Ten-year change in sedentary behavior, moderate-to-vigorous physical activity, cardiorespiratory fitness and cardiometabolic risk: independent associations and mediation analysis. Br J Sports Med. 2016;52:1063-8. http://dx.doi.org/10.1136/bjsports-2016-096083

21. Oliveira RG, Guedes DP. Physical activity, sedentary behavior, cardiorespiratory fitness and metabolic syndrome in adolescents: Systematic review and meta-analysis of observational evidence. PLoS One. 2016;11:e0168503. https://doi.org/10.1371/journal.pone.0168503

22. Wennberg P, Gustafsson PE, Howard B, Wennberg M, Hammarström A. Television viewing over the life course and the metabolic syndrome in mid-adulthood: a longitudinal population-based study. J Epidemiol Community Health. 2014;68:928-33. https://doi.org/10.1136/jech-2013-203504

23. Grøntved A, Ried-Larsen M, Møller NC, Kristensen PL, Wedderkopp N, Froberg K, et al. Youth screen-time behavior is associated with cardiovascular risk in young adulthood: the European Youth Heart Study. Eur J Prev Cardiol. 2014;21:46-56. https://doi.org/10.1177/2047487312454760

24. Wennberg P, Gustafsson PE, Dunstan DW, Wennberg M, Hammarström A. Television viewing and low leisure-time physical activity in adolescence independently predict the metabolic syndrome in mid-adulthood. Diabetes Care. 2013;36:2090-7. https://doi.org/10.2337/dc12-1948

25. Silva DR, Werneck AO, Collings PJ, Fernandes RA, Barbosa DS, Rongue ER, et al. Physical activity maintenance and metabolic risk in adolescents. J Public Health (Oxf.). 2018;40:493-500. https://doi.org/10.1093/pubmed/fdx077

26. Silva D, Werneck AO, Collings P, Tomeleri CM, Fernandes RA, Rongue E, et al. Cardiorespiratory fitness is related to metabolic risk independent of physical activity in boys but not girl from Southern Brazil. Am J Hum Biol. 2016;28:534-8. https://doi.org/10.1002/ajhb.22826

27. Artero EG, Ruiz JR, Ortega FB, España-Romero V, Vicente-Rodríguez G, Molnar D, et al. Muscular and cardiorespiratory fitness are independently associated with metabolic risk in adolescents: the HELENA study. Pediatr Diabetes. 2011;12:704-12. https://doi.org/10.1111/j.1399-5448.2011.00769.x

28. Todendi PF, Valim AR, Reuter CP, Mello ED, Gaya AR, Burgos MS. Metabolic risk in schoolchildren is associated with low levels of cardiorespiratory fitness, obesity, and parents’ nutritional profile. J Pediatr (Rio J). 2016;92:388-93. http://dx.doi.org/10.1016/j.jped.2015.10.007

29. Després JP. Physical activity, sedentary behaviours and cardiovascular health: when will cardiorespiratory fitness become a vital sign? Can J Cardiol. 2016;32:505-13. https://doi.org/10.1016/j.cjca.2015.12.006

30. Gonçalves EC, Silva AS, Nunes HE. Prevalence and factors associated with low aerobic performance levels in adolescents: a systematic review. Curr Pediatr Rev. 2015;11:56-70. https://doi.org/10.2174/1573396311666150501003435

